# Dementia in People With Multiple Sclerosis: A Systematic Review and Meta‐Analysis

**DOI:** 10.1002/brb3.70588

**Published:** 2025-05-30

**Authors:** Omid Mirmosayyeb, Danial Dehghani Firouzabadi, Soroush Oraee, Mohammadreza Alinejadfard, Mohammad Yazdan Panah, Saeed Vaheb, Hamed Ghoshouni, Vahid Shaygannejad

**Affiliations:** ^1^ Isfahan Neurosciences Research Center Isfahan University of Medical Sciences Isfahan Iran; ^2^ Department of Neurology Isfahan University of Medical Sciences Isfahan Iran; ^3^ Student Research Committee Shahid Sadoughi University of Medical Sciences Yazd Iran; ^4^ School of Medicine Shahid Beheshti University of Medical Sciences Tehran Iran; ^5^ Students Research Committee Shahrekord University of Medical Sciences Shahrekord Iran

**Keywords:** Alzheimer's disease, dementia, multiple sclerosis

## Abstract

**Introduction:**

Multiple sclerosis (MS), as an autoimmune demyelinating disorder, is associated with cognitive dysfunction. Dementia can result from severe cognitive dysfunction or other pathways in MS, but the exact mechanisms and prevalence are unknown.

**Objective:**

This review aimed to determine the pooled prevalence and risk of dementia in people with MS (PwMS).

**Design:**

This meta‐analysis was performed in accordance with the guidelines established by the Preferred Reporting Items for Systematic Review and Meta‐Analysis (PRISMA).

**Methods:**

Embase, PubMed, Web of Science, and Scopus were comprehensively searched up to August 29, 2024, to identify observational studies that examined the prevalence or hazard ratio (HR) of dementia among PwMS. This meta‐analysis used a random‐effects model to calculate the pooled prevalence and risk of dementia among PwMS, where the prevalence rate and HR were the main metrics for effect size.

**Results:**

Ten studies, including a total of 37,831 PwMS, estimated the prevalence of dementia in PwMS to be 5.31% (*I* ^2^ = 99.2%, 95% CI: 2.25%–11.98%). In addition, a meta‐analysis of four studies assessed the HR of dementia among PwMS, revealing a pooled HR of 1.67 (*p* < 0.01, *I* ^2^ = 73.5%, 95% CI: 1.31–2.13).

**Conclusion:**

While dementia is not a common feature of MS, PwMS still have a significantly higher risk of developing it, compared to healthy indiviuals. However, the considerable variability across studies indicates that these estimates should be interpreted with caution, as inconsistencies in research approaches may have influenced the results. These findings warrant further validation.

## Graphical abstract

In this study, the overall prevalence and risk of dementia in PwMS was examined. It was shown that MS may increase the risk of dementia by up to 67% through two mechanisms that cause atrophy of white and gray matter.

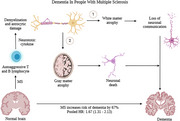

## 1 Introduction

Multiple sclerosis (MS) is a chronic autoimmune disorder marked by damage to the myelin sheath within the central nervous system (CNS) (Lubetzki and Stankoff 2014, Coutinho Costa et al. 2023). MS manifests itself with a variety of clinical features, including motor dysfunction, sensory disturbances, and autonomic irregularities (Javalkar et al. 2016). In addition, cognitive dysfunction is also a prevalent finding, with estimates ranging from 30% to 70%, depending on the definition used (Benedict et al. 2020). These dysfunctions are attributed to the disruption of white matter connections and the atrophy of gray matter in people with multiple sclerosis (PwMS) (Engl et al. 2020).

Reduced speed of processing information and difficulties with acquiring new knowledge are central aspects of cognitive dysfunction in MS (Guimarães and Sá 2012), which may be distinct from patterns of dementia and cognitive dysfunction observed in other pathologies, including Alzheimer's disease (AD) (Vidorreta‐Ballesteros et al. 2024). Nevertheless, as patients grow older, other forms of neurodegenerative dementia may coexist with cognitive deficits specific to MS, making it more difficult to distinguish between MS‐related cognitive decline and dementia (Jakimovski et al. 2019).

Dementia refers to a broad category of acquired cognitive decline spanning multiple domains, which significantly disrupts daily functioning, distinguishing it from cognitive impairment, where deficits may not impair occupational or social capacities (Cipriani et al. 2020, Kumar et al. 2024). It is marked by deficiencies in memory, language, reasoning, cognitive processes, and the ability to perform everyday tasks (Westervelt 2015). Thus, dementia is not regarded as a specific disease; rather, it is a syndrome associated with a range of neurological disorders.

MS is one of the CNS pathologies that dementia may be related to (Boukhvalova et al. 2023). MS can exacerbate specific pathologies related to AD and may cause cognitive decline severe enough to be classified as dementia (Willumsen et al. 2022). Moreover, inflammation, neurodegeneration, and disrupted cellular metabolism, which are prominent in MS, may lead to dementia and dementia‐like symptoms, as well as exacerbate beta‐amyloid accumulation, which is prominent in AD (Livingston et al. 2020). However, the exact relationship between MS and dementia, including specific types of dementia like AD, is still not completely understood, and it remains unclear whether MS can be classified as a risk factor for dementia.

Given the increased life expectancy of PwMS in recent years (Willumsen et al. 2022), it is important to evaluate complications that are more prevalent in older populations, including dementia. In addition, establishing the occurrence of dementia in MS patients is crucial to help practitioners address it properly (Livingston et al. 2020). With this in mind, this review aimed to determine the pooled prevalence and risk of dementia in PwMS.

## 2 Methods

This systematic review and meta‐analysis was conducted in accordance with the Preferred Reporting Items for Systematic Reviews and Meta‐Analyses (PRISMA) guidelines (Page et al. 2021), with a PROSPERO registration ID of CRD42024611529.

### 2.1 Search Strategy

The systematic search was conducted from inception to August 29, 2024, through the following databases: PubMed, Embase, Scopus, and Web of Science. The search strategy included MeSH terms and text words related to MS and dementia. In addition, the reference lists of relevant studies and review articles were meticulously examined to ensure the exhaustive incorporation of applicable research. The search syntax for each database is detailed in Supporting Information.

### 2.2 Eligibility Criteria

Studies adhering to the following criteria were incorporated: (1) English articles; (2) Observational studies such as cross‐sectional, case‐control, and cohorts; (3) Involving adult participants (aged 18 years or older) diagnosed with MS according to the established diagnostic criteria at the time of the study; (4) Reporting the prevalence or hazard ratio (HR) of dementia among PwMS; (5) The diagnosis of dementia was based on medical records, Diagnostic and Statistical Manual of Mental (DSM) criteria, or International Classification of Diseases (ICD) codes.

Studies that conformed to the following criteria were excluded: (1) non‐English articles, Duplicate studies, and Studies with insufficient or unavailable data; (2) Case reports/case series, randomized clinical trials; (3) In vitro and animal studies; (4) Reviews, conference papers, letters to editor, and protocols, (5) Studies have identified dementia as a form of cognitive impairment.

### 2.3 Study Selection

Two reviewers (D.D. and S.V.) independently screened the titles and abstracts of the articles. If an abstract appeared relevant to the review question, full‐text articles were evaluated for eligibility according to the established inclusion and exclusion criteria. Disagreements were resolved through discussion or consulting a third reviewer (O.M.).

### 2.4 Data Extraction

Two reviewers (D.D. and S.V.) independently extracted data from the included studies. This process involved gathering information such as first author, country, year of publication, study design, Expanded Disability Status Scale (EDSS) scores, sample size, demographics, disease duration, the frequency of dementia in PwMS, type of dementia, diagnostic methods for dementia, and the HR of dementia in PwMS. In cases where the required data were unavailable, the study authors were contacted via email for additional information. If neither was possible, the study was not considered in the meta‐analysis.

### 2.5 Quality Assessment

Two reviewers (H.G. and M.Y.P.) independently, using the Newcastle‐Ottawa Scale (NOS) (Modesti et al. 2016), assessed the quality of the included studies. This scale assesses key aspects of observational studies, including sample selection, comparability between case and control groups, and exposure assessment. A third researcher (O.M.) resolved any disagreements.

### 2.6 Data Analysis

All statistical analyses were carried out using R software version 4.3.3 (R Core Team 2023). A random‐effects model was employed in all analyses to account for methodological variations between studies. Statistical heterogeneity was evaluated using Cochran's *Q* test and the *I* ^2^ index, with higher *I* ^2^ values indicating greater heterogeneity (Hardy and Thompson 1998). Publication bias was assessed using Egger's and Begg's tests and visual examination of funnel plots. The prevalence of dementia among PwMS was estimated using a proportional meta‐analysis, pooling the proportions of dementia cases across studies. To assess the risk of dementia among PwMS, the HRs from the included studies were combined with a random‐effects model. To identify potential sources of heterogeneity, subgroup analyses and meta‐regression were conducted. In addition, to adjust the presence of publication bias and evaluate funnel plot asymmetry, the trim‐and‐fill method was applied.

## 3 Results

### 3.1 Study Selection

**FIGURE 1:**
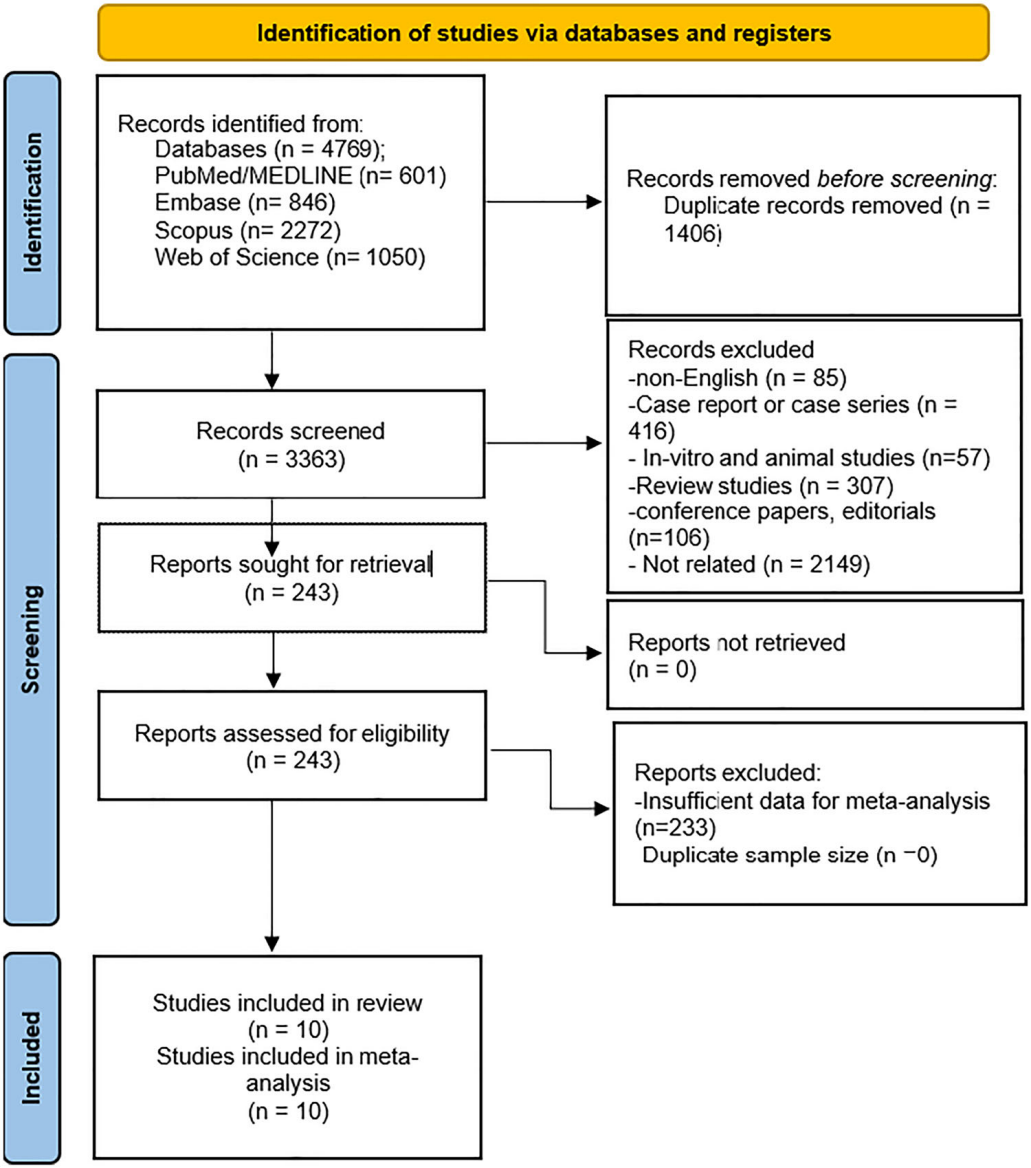
PRISMA flow diagram of the study process.

Our initial search identified 4769 potential studies for inclusion. After removing the duplicates, 3363 potential studies were found for inclusion. During the screening process, 3120 articles were excluded based on the established inclusion and exclusion criteria. Then, 243 unique full‐text articles were evaluated. After removing articles with insufficient data, 10 studies were ultimately included. Figure 1 presents additional details of the study selection process.

### 3.2 Study Characterization

The studies were published between 1994 and 2024, with designs including cross‐sectional (*n* = 4), cohort (*n* = 5), and case‐control (*n* = 1). Most of the studies were from the USA (*n* = 4, 40%) (Buchanan et al. 2005, Demakis et al. 2009, Mahmoudi et al. 2022, Fleming et al. 2024), followed by Austria (Zinganell et al. 2024), Taiwan (Kang et al. 2010), South Korea (Cho et al. 2023), the Netherlands (Nuyen et al. 2006), Germany (Möller et al. 1994), and the United Kingdom (Chou et al. 2020) (*n* = 1 per country). Sample sizes ranged from 25 to 20,561 participants, and the total number of PwMS across all studies was 37,831. Among the included studies, the study population predominantly consisted of females (total female = 23,248; 61%). The total mean age of the PwMS was 57.36 years (SD = 10.19). EDSS and disease duration of PwMS were not reported. However, AD and related dementias (ADRD) were reported in two studies (*n* = 6103). Diagnostic Assessment Tools for dementia were ICD‐9 (*n* = 5), ICD‐10 (*n* = 4), and ICPC (*n* = 1). More details of the main characteristics of the included studies are outlined in Table 1.

**TABLE 1:** The principal features of the included studies.

First author year country	Study design	PwMS F:M age; mean (SD)	MS type (*n*)	EDSS	Disease duration (years); mean (SD)	Types of dementia (*n*)	Diagnostic assessment of dementia	Prevalence of dementia (%)	Major findings	Quality assessment
N.H. Fleming 2024 USA (Fleming et al. 2024)	Cohort	4048 263:3821 65.9;(7)	NR	NR	NR	NR	ICD‐9 and ICD‐10	16.67	PWMS had a higher risk of dementia compared to controls^a^	7
A. Zinganell 2024 Austria (Zinganell et al. 2024)	Cohort	1200 857:443 45;(37–52)^b^	RRMS:924 PPMS:191 SPMS:52 CIS:33	NR	NR	NR	Database	2.75	Dementia was associated with reaching EDSS 6 and EDSS 7	8
E.B. Cho 2023 South Korea (Cho et al. 2023)	Cohort	1347 782:565 56.2;(10.6)	NR	NR	NR	ADRD:78 Vascular:15 Other:8	ICD‐10	7.50	In PWMS, the risk of developing any form of dementia was higher compared to controls^c^	8
E. Mahmoudi 2022 USA (Mahmoudi et al. 2022)	Cohort	6025 4268:1757 NR	NR	NR	NR	ADRD	ICD‐9‐CM or ICD‐10‐CM	25	PWMS revealed a higher risk for early‐onset ADRD diagnosis^d^	8
I.J. Chou 2020 United Kingdom (Chou et al. 2020)	Cohort	2526 1790:736 45.03;(12.37)	NR	NR	NR	NR	Data base	0.51	The risk of dementia was not significantly higher among PWMS compared with controls^e^	8
J.H. Kang 2010 Taiwan (Kang et al. 2010)	Case‐control	898 NR NR	NR	NR	NR	NR	ICD‐9‐CM	2.23	The risk of developing dementia was significantly higher among MS patients	7
G.J. Demakis 2009 USA (Demakis et al. 2009)	Cross‐sectional	924 665:259 57.5;(13.5)	NR	NR	NR	Dementia other than Alzheimer's disease	ICD‐9‐CM	12.12	Dementia was more prevalent among MS‐Neuro patients compared to MS‐Comb patients	6
J. Nuyen 2006 Netherlands (Nuyen et al. 2006)	Cross‐sectional	241 168:73 49.1(12.7)	NR	NR	NR	NR	ICPC	0.00	None of the individuals in the cohort displayed any signs or symptoms of dementia	7
R.J. Buchanan 2005 USA (Buchanan et al. 2005)	Cross‐sectional	20,561 14,437:6124 NR	NR	NR	NR	NR	ICD‐9‐CM	10.87	A notable portion of admitted PWMS had dementia	6
A. Möller 1994 Germany (Möller et al. 1994)	Cross‐sectional	25 18:7 37(NR)	NR	3.3;(1–7.5)^f^	7.7;(1–25)^f^	NR	SIDAM(ICD‐10)	8	Dementia was prevalent in the study population	6

Abbreviations: ADRD: Alzheimer's disease and related dementias, CIS: clinically isolated syndrome, EDSS: expanded disability status scale, ICD: international classification of disease, ICPC: international classification of primary care, MS‐Comb: multiple sclerosis with both neurological and psychiatric co‐morbidities, MS‐Neuro: multiple sclerosis with neurological co‐morbidities, NR: not reported or unavailable data, PPMS: primary progressive multiple sclerosis, PwMS: people with multiple sclerosis, RRMS: relapsing‐remitting multiple sclerosis, SIDAM: Structured Interview for the Diagnosis of Alzheimer Dementia and Dementias of other Aetiology, SPMS: secondary progressive multiple sclerosis.

^a^ Hazard ratio adjusted for sex, age, race and ethnicity, geographic latitude, depression, tobacco use, substance abuse, stroke, epilepsy, Parkinson's disease, obesity, diabetes, cardiac disease, and dyslipidemia.

^b^ Median (range).

^c^ Hazard ratio adjusted for age, sex, household income, and presence of diabetes mellitus, hypertension, or dyslipidemia.

^d^ Hazard ratio adjusted for demographics, socioeconomic, and comorbidities.

^e^ Hazard ratio adjusted for BMI class, smoking status, and alcohol consumption.

^f^ Mean (range).

**Median (IQR).

^z^Z‐score.

### 3.3 Prevalence and Risk of Dementia Among PwMS

**FIGURE 2:**
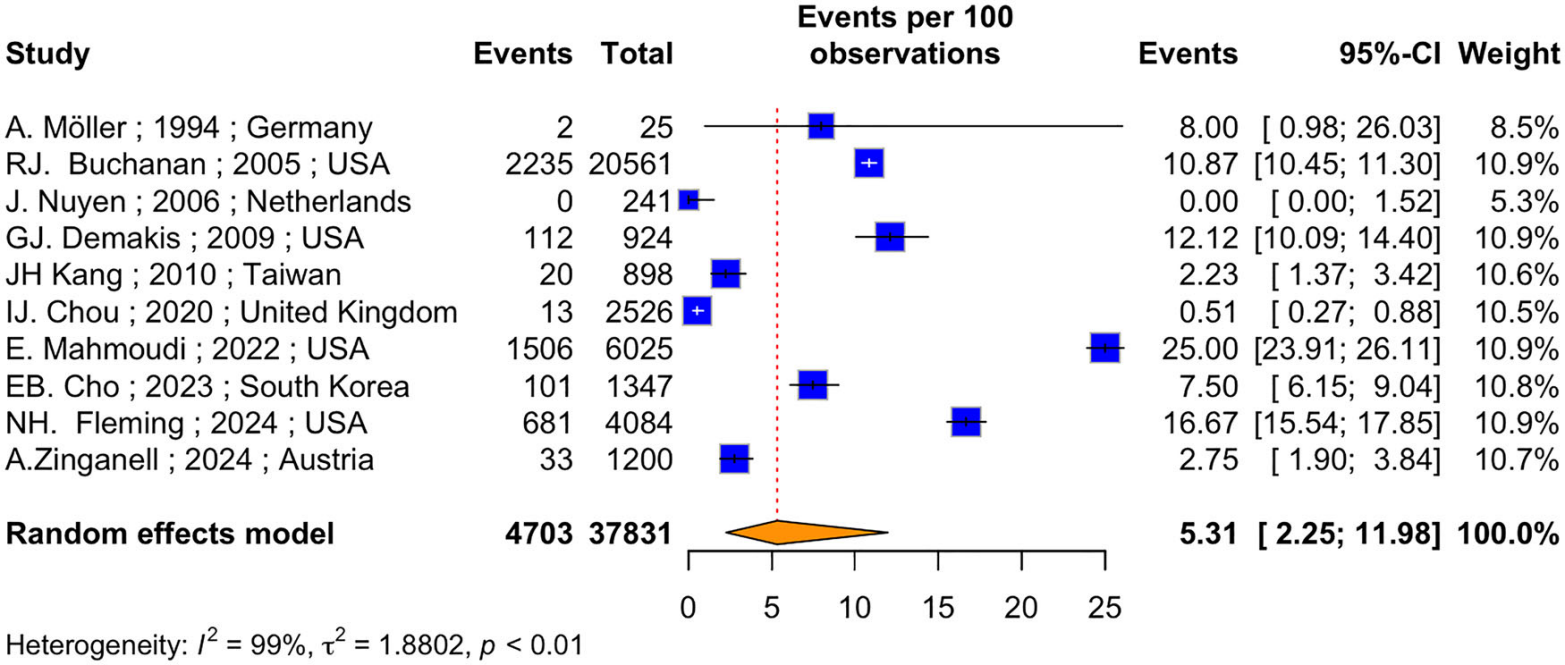
Forest plot of the pooled prevalence of dementia in PwMS.

**FIGURE 3:**
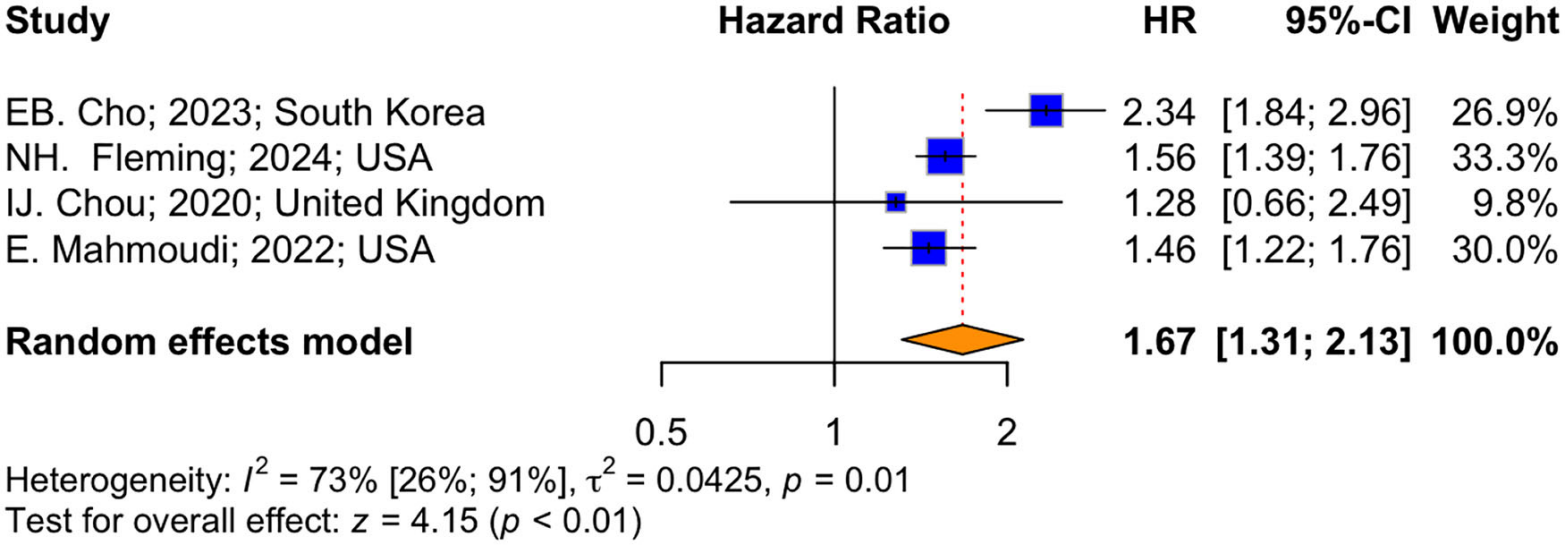
Forest plot of the pooled hazard ratio of dementia in PwMS.

The meta‐analysis on 10 studies with a total of 37,831 patients and 4703 dementia cases, estimated the prevalence of dementia in PwMS to be 5.31% (*I* ^2^ = 99.2%, 95% CI: 2.25%–11.98%) (Figure 2). In addition, a meta‐analysis on four studies, assessing the HR of dementia among PwMS, revealed a pooled HR of 1.67 (*p* < 0.01, *I* ^2^ = 73.5%, 95% CI: 1.31–2.13) based on a random‐effects model (Figures 3).

### 3.4 Alzheimer's Disease in PwMS

Two studies assessed AD in PwMS (Mahmoudi et al. 2022, Cho et al. 2023). Due to the limited number of studies, a meta‐analysis was not conducted. PwMS had a significantly higher risk of developing AD and ADRD, especially at younger ages. In adults aged 45–64 with MS, the incidence of early‐onset ADRD was 1.4%, which is seven times higher than the 0.2% incidence in individuals without MS. In adults aged 65 and older, the incidence of the condition was 4.0% among PwMS, compared to 3.3% in those without MS. HRs further emphasize this increased risk, with an adjusted HR of 2.23 across all ages and up to 4.49 for early‐onset ADRD in middle‐aged adults. However, given that these findings are based on only two studies, further research is needed to confirm these associations and understand the underlying mechanisms.

### 3.5 Subgroup Analysis

The subgroup analysis based on the continent revealed that studies conducted in America reported the highest prevalence of dementia among MS patients at 15.51% (95% CI:10.52–22.27]), followed by Asia at 4.18% (95% CI: 1.24–13.15) and Europe at 1.49% (95% CI:0.35–6.09), and this difference was statistically significant (*p* = 0.0011) (Figure S1). In addition, our findings showed that cross‐sectional studies reported a higher prevalence of dementia as compared to cohort studies (10.97%, 95% CI: 10.32–11.66) vs. 5.76%, 95% CI: 1.42–20.61), and this difference was statistically significant (*p* < 0.001) (Figure S2).

Moreover, Studies utilizing the ICD reported a higher prevalence of 10.09% (95% CI: 5.54–17.66) compared to 0.90% (95% CI: 0.21–3.76) for studies using other diagnostic criteria and the difference between diagnostic criteria was statistically significant (*p* = 0.0020) (Figure S3). Age variations were also analyzed, with studies including participants aged 50 and above reporting a higher dementia event rate of 11.63% (95% CI: 7.28–18.06) compared to 4.05% (95% CI: 1.33–11.62) among those younger than 50 and the difference between age groups was statistically significant (*p* = 0.0061) (Figure S4). However, subgroup analysis revealed that study quality and sample size variation did not significantly impact dementia prevalence (Figures S5 and S6).

### 3.6 Publication Bias

**FIGURE 4:**
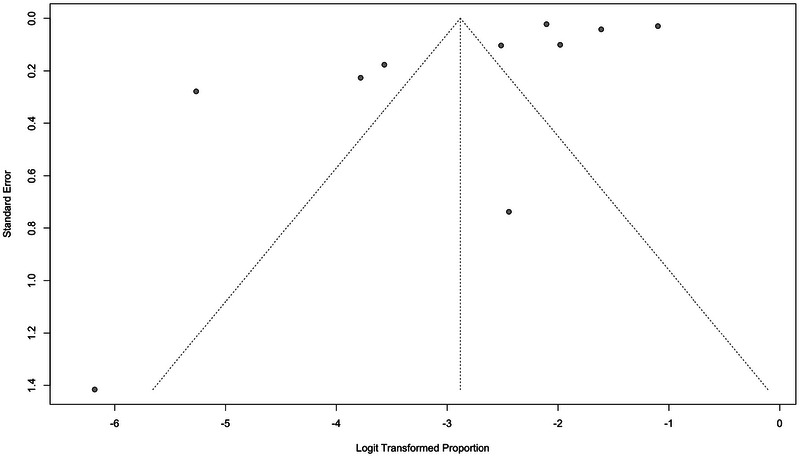
Funnel plot of studies assessing the prevalence of dementia in PwMS.

**FIGURE 5:**
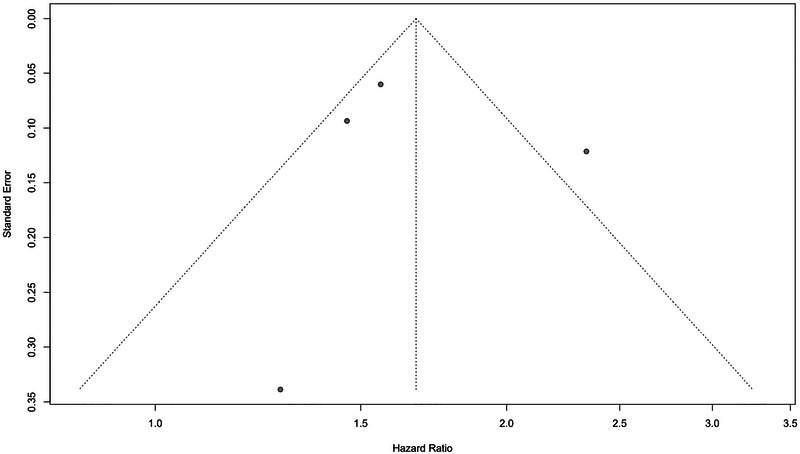
Funnel plot of studies assessing the hazard ratio of dementia in PwMS.

Publication bias was assessed for meta‐analyses using Egger's and Begg's tests, in addition to visual inspection of funnel plots. For the meta‐analysis of dementia prevalence among PwMS, Egger's test (*p* = 0.2416) and Begg's test (*p* = 0.5312) did not provide evidence of significant publication bias. Similarly, for the meta‐analysis of the HR of dementia among PwMS, Egger's test (*p* = 0.8216) and Begg's test (*p* = 0.4969) indicated no signs of publication bias. In addition, the funnel plots for both meta‐analyses are presented in Figures 4 and 5.

The trim‐and‐fill analysis adjusted for publication bias by imputing five missing studies, yielding a pooled prevalence of 14.63% (95% CI:5.34%–34.24%) employing a random‐effects model. Although the adjustment addressed funnel plot asymmetry, the significant heterogeneity (*I* ^2^ = 99.1%, *p* < 0.001) requires cautious interpretation of the pooled estimate (Figure S7).

### 3.7 Meta Regression

A meta‐regression analysis was conducted to explore potential factors that might influence the prevalence of dementia. The results indicated that none of the variables examined—such as the year of publication (estimate = 0.0100, *p* = 0.8427), sample size (estimate = 0.0001, *p* = 0.3135), study quality (estimate = −0.4305, *p* = 0.4326), age (estimate = 0.0864, *p* = 0.1099), or sex ratio (estimate = −0.4562, *p* = 0.4852)—had a statistically significant impact on the overall prevalence of dementia. Detailed results of the meta‐regression can be found in Table S2 and Figures S8–S12.

### 3.8 Quality Assessment

According to the quality assessment, three studies scored 6, whereas seven studies received scores greater than 6 (Table 1). The mean NOS score across all studies was 7.1 (SD = 0.83), suggesting that the methodological quality of the included studies was high.

## 4 Discussion

This review estimated the pooled prevalence and HR of dementia in PwMS. We found that an estimated 5.31% of PwMS suffer from dementia, with significant variations based on continent, study design, diagnostic criteria, and age. The highest prevalence was reported in America (15.51%), and cross‐sectional studies, ICD‐based diagnoses, and older age groups were associated with higher rates. However, study quality and sample size had no significant impact. Our meta‐regression found no significant effect of publication year, sample size, study quality, age, or sex ratio on prevalence. Also, we found that the HR of dementia in PwMS is 1.67 compared to the healthy population, indicating that there is a 67% higher chance of MS patients being diagnosed with dementia compared to the general population.

The pathophysiology of dementia in PwMS is complex and involves several key mechanisms (Chiang et al. 2022). MS is mainly defined by immune‐mediated demyelination and neurodegeneration within the CNS, resulting in impaired neuronal communication (Cavallo 2020). This loss of myelin leads to axonal damage and a reduction in the integrity of white matter, which are critical pathways for efficient brain functioning and cognition (Friese et al. 2014, Lin et al. 2023). The inflammatory processes in MS, including the activation of microglia and astrocytes, contribute to neurodegenerative changes and are associated with the release of neurotoxic cytokines (Mado et al. 2023).

Moreover, MS‐related neurodegeneration may lead to cortical and subcortical gray matter atrophy, both of which are linked to cognitive dysfunction and increased dementia risk (Fuchs et al. 2018, van de Mortel et al. 2021, Bussas et al. 2022). Oxidative stress and mitochondrial dysfunction further exacerbate neuronal injury (Su et al. 2013). In addition, pathways like mTOR and the involvement of genes such as APOE‐ε4 have also been implicated in the progression of cognitive deficits in MS patients (Engel et al. 2020, Van Skike et al. 2020, Vakrakou et al. 2022). These pathways underscore the interplay between inflammation, cellular metabolism, and neurodegeneration in the development of dementia within the MS population. Finally, MS lesions themselves may play a role, as previous studies have shown that cognitive impairment in PwMS is correlated with the number of cortical and white matter MS lesions (Mike et al. 2011, Curti et al. 2018). Nonetheless, the magnitude of the impact and whether it would lead to cognitive impairment severe enough to be diagnosed as dementia require further study.

AD, the most common form of dementia, has also been observed in PwMS (Cho et al. 2023). While cognitive impairment in MS primarily affects processing speed and executive functioning, AD is generally associated with deficits in episodic memory, severe forgetfulness, and difficulties in recognizing familiar objects or faces (Drew et al. 2008, Karantzoulis and Galvin 2011). Research indicates that neuroinflammation is a critical factor in both conditions. In MS, T and B lymphocyte‐mediated immune responses activate microglia, contributing to the development of demyelinated lesions and neuronal damage (Karantzoulis and Galvin 2011). Similar mechanisms, such as microglial activation and inflammation, are also implicated in the progression of AD (Miao et al. 2023), suggesting that the inflammatory milieu in MS might accelerate the pathogenesis of Alzheimer's‐like dementia (Kinney et al. 2018).

Diagnosing AD in a clinical setting is challenging, as a definitive confirmation requires a postmortem autopsy (Weller and Budson 2018). This complicates the differentiation of whether cognitive dysfunction in PwMS is solely attributed to the condition itself or is influenced by coexisting AD. Biomarkers offer a way to sort it out, with tools like PET imaging and CSF analysis stepping in to clarify things (Londoño et al. 2022). The 18F‐FDG PET scans reveal a scattered or patchy drop‐in brain activity in MS, tied to damaged nerve fibers, while AD shows a distinct pattern of reduced activity in the temporoparietal and posterior cingulate areas (Londoño et al. 2022). CSF tests can also provide more insight. In AD, Aβ1‐42 levels are typically low, while tau levels are high. In contrast, MS usually presents with normal Aβ1‐42 levels and only a slight increase in tau, unless AD itself is present (Londoño et al. 2022).

Our study found a much lower prevalence of dementia compared to the high rate of cognitive impairment in PwMS (Benedict et al. 2020). This discrepancy is primarily due to the more restrictive definition of dementia. Cognitive impairment is broadly defined, with any deficit in a single cognitive domain being classified as cognitive impairment (Anand and Schoo 2025). In contrast, dementia requires significant impairment in at least two cognitive domains, accompanied by substantial disruption in occupational or social functioning. This diagnosis is confirmed through clinical evaluations, neuropsychological testing, and functional assessments (Arvanitakis et al. 2019).

Our subgroup analyses revealed that the highest prevalence of dementia among PwMS was observed in studies conducted in America (15.51%), potentially attributable to variations in healthcare access, diagnostic practices, or population‐specific factors such as comorbidities or genetic predispositions (Fratiglioni et al. 1999, Llibre‐Guerra et al. 2024). In contrast, lower prevalence estimates reported in studies from other continents may reflect underdiagnosis, differences in MS disease progression, or methodological inconsistencies across investigations.

Study design also influenced prevalence estimates, with cross‐sectional studies reporting higher rates compared to longitudinal studies. This disparity may stem from cross‐sectional designs capturing a greater proportion of advanced or severe cases at a single time point, whereas longitudinal studies might underestimate early dementia due to limited follow‐up duration or participant attrition.

Moreover, the choice of diagnostic criteria has a big impact on prevalence numbers, with studies using ICD‐based methods tending to show higher rates than those relying on other approaches. The ICD appears to be more flexible, identifying more cases compared to the stricter DSM or detailed neuropsychological tests. Interestingly, a comparison of ICD‐10 and DSM‐IV dementia diagnoses showed perfect agreement (kappa = 1.0), almost as if they were in complete harmony. However, ICD‐10 allows some room for interpretation, particularly when assessing complex cognitive skills such as abstract thinking, judgment, or problem‐solving. This flexibility can lead to inconsistent diagnoses and misclassification bias, distorting prevalence estimates and complicating dementia research (Naik and Nygaard 2008). Future studies should incorporate thorough clinical assessments for more accuracy.

Our subgroup analysis revealed striking age‐related disparities. Among PwMS over 50, dementia prevalence reached 11.63%, while it dropped to just 1.49% in those under 50. In contrast, the general population typically shows dementia prevalence below 3% for individuals under 70 (Prince et al. 2013). This suggests that PwMS not only face higher rates at younger ages but also experience a sharper rise in prevalence as they age compared to their peers without MS. For instance, the 11.63% prevalence in PwMS over 50 far exceeds what's typical for that age range in the general population, where rates don't climb significantly until later decades. These findings support the notion that MS may accelerate or exacerbate age‐related cognitive decline, possibly due to chronic neuroinflammation, white matter damage, and cortical atrophy, which may accumulate over time in this population.

Overall, these findings highlight the importance of considering geographic, methodological, and demographic variables when assessing dementia prevalence in PwMS. The increased prevalence in specific subgroups, particularly older individuals and those in certain regions, underscores the need for targeted cognitive screening and the implementation of standardized diagnostic protocols in clinical settings. Future research should examine the underlying causes of regional disparities and explore whether MS‐specific mechanisms contribute to dementia risk beyond the effects of normative aging.

This meta‐analysis is the first one to assess dementia in PwMS. Our findings are clinically significant, as they can guide practitioners to be more vigilant about dementia in PwMS and encourage patients to take proactive steps, such as adopting lifestyle changes or participating in cognitive rehabilitation, both of which have demonstrated promising outcomes (Kudlicka et al. 2023, Ornish et al. 2024). PwMS regularly visits neurologists, enabling earlier detection of changes in cognitive function. However, the pathophysiology of dementia in PwMS remains poorly understood, complicating efforts to ascertain the effects of treatments on its progression. In addition, disease‐modifying therapies (DMTs), which have emerged relatively recently, show uncertain long‐term impacts on cognitive decline. While studies suggest DMTs may have small to moderate positive effects on cognitive test performance, particularly in processing speed, no significant differences in cognitive improvement have been observed between DMT types (Landmeyer et al. 2020, Kania et al. 2023). All in all, the clinical implications for PwMS warrant careful consideration. Routine cognitive screening, especially as patients age, could facilitate early detection. Future research should prioritize establishing standardized screening protocols and evaluating the long‐term benefits and cost‐effectiveness of novel treatments, as well as the role of early cognitive monitoring in this context.

This review had some limitations. The very high heterogeneity (*I* ^2^ = 99.2%) in our pooled prevalence estimate shows significant differences across the studies that our subgroup analyses couldn't explain. We looked at factors like geographic region, study design, diagnostic criteria, and age, but none reduced the variability much. These findings suggest that unexamined factor such as clinical features of MS, including disease duration, subtype, and severity of disability, methodological heterogeneity in cognitive evaluation tools, and demographic variables extending beyond age and sex likely contribute to the variability in dementia prevalence observed in people with MS. Interactions between these factors might also play a role, though our analysis couldn't detect them.

This persistent heterogeneity limits our ability to pinpoint the true prevalence of dementia in PwMS. While we found pattern, higher prevalence in American studies or with ICD‐based diagnoses, the pooled estimate of 5.31% is a rough average with a wide range of possible values. It shouldn't be taken as a precise figure for all PwMS. The results call for more consistent research methods and caution against broad generalizations. In short, the high heterogeneity across subgroups shows dementia in MS is complex, influenced by factors this study could not fully capture. Future studies using individual patient data could better address these variables and refine dementia risk estimates for this population.

## 5 Conclusion

While dementia is not a common feature of MS, PwMS still have a significantly higher risk of developing it compared to healthy controls, highlighting the importance of early recognition and monitoring of cognitive decline and dementia in this group. However, the considerable variability across studies indicates that these estimates should be interpreted with caution, as inconsistencies in research approaches may have influenced the results. ADRD are also more prevalent in PwMS compared to the healthy population, although additional studies are warranted to confirm these findings.

## Author Contributions

**Omid Mirmosayyeb**: conceptualization, methodology, supervision, validation, investigation, writing – review and editing, project administration. **Danial Dehghani Firouzabadi**: data curation. **Soroush Oraee**: writing – original draft. **Mohammadreza Alinejadfard**: writing – original draft. **Mohammad Yazdan Panah**: conceptualization, methodology, formal analysis, visualization, investigation, writing – review and editing. **Saeed Vaheb**: data curation, writing – review and editing, investigation, validation. **Hamed Ghoshouni**: formal analysis, software. **Vahid Shaygannejad**: conceptualization, methodology, investigation, writing – review and editing.

## Ethics Statement

The authors have nothing to report.

## Conflicts of Interest

The authors declare no conflicts of interest.

### Peer Review

The peer review history for this article is available at https://publons.com/publon/10.1002/brb3.70588.

## Supporting information



Supplementary Materials

## Data Availability

This is a systematic review article and all relevant data are within the manuscript and tables.
