# Supplementary material for: Dementia in People With Multiple Sclerosis: A Systematic Review and Meta‐Analysis
Source: Brain Behav. 2025 May 30;15(6):e70588. doi: 10.1002/brb3.70588 (PMC12123099; doi:10.1002/brb3.70588)
Supplement: Supplementary file 1 — Supplementary Materials [file BRB3-15-e70588-s001.docx]

| Syntax  **Supplementary Material: Syntax of queries used to search PubMed/Medline, Scopus, Web of Science, and Embase** |  |
| --- | --- |
| Pubmed/MEDLINE | |
| (("Dementias"[tiab] OR "Amentia"[tiab] OR "Amentias"[tiab] OR "Senile Paranoid Dementia"[tiab] OR "Dementias, Senile Paranoid"[tiab] OR "Paranoid Dementia, Senile"[tiab] OR "Paranoid Dementias, Senile"[tiab] OR "Senile Paranoid Dementias"[tiab] OR "Familial Dementia"[tiab] OR "Dementia, Familial"[tiab] OR "Dementias, Familial"[tiab] OR "Familial Dementias"[tiab] OR "Dementia, AIDS Dementia Complex"[tiab] OR "Alzheimer Disease"[tiab] OR "Aphasia, Primary Progressive"[tiab] OR "Primary Progressive Nonfluent Aphasia"[tiab] OR "Creutzfeldt-Jakob Syndrome"[tiab] OR "Dementia, Vascular"[tiab] OR "CADASIL"[tiab] OR "Dementia, Multi-Infarct"[tiab] OR "Diffuse Neurofibrillary Tangles with Calcification"[tiab] OR "Frontotemporal Lobar Degeneration"[tiab] OR "Frontotemporal Dementia"[tiab] OR "Primary Progressive Nonfluent Aphasia"[tiab] OR "Huntington Disease"[tiab] OR "Kluver-Bucy Syndrome"[tiab] OR "Lewy Body Disease"[tiab] OR "Mixed Dementias"[tiab]) AND ("Sclerosis, Multiple"[tiab] OR "MS (Multiple Sclerosis)"[tiab] OR "Sclerosis, Disseminated"[tiab] OR "Disseminated Sclerosis"[tiab] OR "Multiple Sclerosis, Acute Fulminating"[tiab] OR "multiple sclerosis"[tiab])) | 601 |
| Scopus | |
| ( ( "Dementias"[tiab] OR "Amentia"[tiab] OR "Amentias"[tiab] OR "Senile Paranoid Dementia"[tiab] OR "Dementias, Senile Paranoid"[tiab] OR "Paranoid Dementia, Senile"[tiab] OR "Paranoid Dementias, Senile"[tiab] OR "Senile Paranoid Dementias"[tiab] OR "Familial Dementia"[tiab] OR "Dementia, Familial"[tiab] OR "Dementias, Familial"[tiab] OR "Familial Dementias"[tiab] OR "Dementia, AIDS Dementia Complex"[tiab] OR "Alzheimer Disease"[tiab] OR "Aphasia, Primary Progressive"[tiab] OR "Primary Progressive Nonfluent Aphasia"[tiab] OR "Creutzfeldt-Jakob Syndrome"[tiab] OR "Dementia, Vascular"[tiab] OR "CADASIL"[tiab] OR "Dementia, Multi-Infarct"[tiab] OR "Diffuse Neurofibrillary Tangles with Calcification"[tiab] OR "Frontotemporal Lobar Degeneration"[tiab] OR "Frontotemporal Dementia"[tiab] OR "Huntington Disease"[tiab] OR "Kluver-Bucy Syndrome"[tiab] OR "Lewy Body Disease"[tiab] OR "Mixed Dementias"[tiab] )  AND ( "Sclerosis, Multiple"[tiab] OR "MS (Multiple Sclerosis)"[tiab] OR "Sclerosis, Disseminated"[tiab] OR "Disseminated Sclerosis"[tiab] OR "Multiple Sclerosis, Acute Fulminating"[tiab] OR "multiple sclerosis"[tiab] ) ) | 2272 |
| Web of Science | |
| TS=("Dementias" OR "Amentia" OR "amential" OR "Senile Paranoid Dementia" OR "Dementias, Senile Paranoid" OR "Paranoid Dementia, Senile" OR "Paranoid Dementias, Senile" OR "Senile Paranoid Dementias" OR "Familial Dementia" OR "Dementia, Familial" OR "Dementias, Familial" OR "Familial Dementias" OR "Dementia, AIDS Dementia Complex" OR "Alzheimer Disease" OR "Aphasia, Primary Progressive" OR "Primary Progressive Nonfluent Aphasia" OR "Creutzfeldt-Jakob Syndrome" OR "Dementia, Vascular" OR "CADASIL" OR "Dementia, Multi-Infarct" OR "Diffuse Neurofibrillary Tangles with Calcification" OR "Frontotemporal Lobar Degeneration" OR "Frontotemporal Dementia" OR "Primary Progressive Nonfluent Aphasia" OR "Huntington Disease" OR "Kluver-Bucy Syndrome" OR "Lewy Body Disease" OR "Mixed Dementias") | 1050 |
| Embase | |
| ('Dementias':ti,ab OR 'Amentia':ti,ab OR 'Amentias':ti,ab OR 'Senile Paranoid Dementia':ti,ab OR 'Dementias, Senile Paranoid':ti,ab OR 'Paranoid Dementia, Senile':ti,ab OR 'Paranoid Dementias, Senile':ti,ab OR 'Senile Paranoid Dementias':ti,ab OR 'Familial Dementia':ti,ab OR 'Dementia, Familial':ti,ab OR 'Dementias, Familial':ti,ab OR 'Familial Dementias':ti,ab OR 'Dementia, AIDS Dementia Complex':ti,ab OR 'Alzheimer Disease':ti,ab OR 'Aphasia, Primary Progressive':ti,ab OR 'Primary Progressive Nonfluent Aphasia':ti,ab OR 'Creutzfeldt-Jakob Syndrome':ti,ab OR 'Dementia, Vascular':ti,ab OR 'CADASIL':ti,ab OR 'Dementia, Multi-Infarct':ti,ab OR 'Diffuse Neurofibrillary Tangles with Calcification':ti,ab OR 'Frontotemporal Lobar Degeneration':ti,ab OR 'Frontotemporal Dementia':ti,ab OR 'Primary Progressive Nonfluent Aphasia':ti,ab OR 'Huntington Disease':ti,ab OR 'Kluver-Bucy Syndrome':ti,ab OR 'Lewy Body Disease':ti,ab OR 'Mixed Dementias':ti,ab)  AND  ('Sclerosis, Multiple':ti,ab OR 'MS (Multiple Sclerosis)':ti,ab OR 'Sclerosis, Disseminated':ti,ab OR 'Disseminated Sclerosis':ti,ab OR 'Multiple Sclerosis, Acute Fulminating':ti,ab OR 'multiple sclerosis':ti,ab) | 846 |
|  | |

**Supplementary Table 2. Meta Regression Analysis**

| Covariate | Meta‐regression | | | | | |
| --- | --- | --- | --- | --- | --- | --- |
|  | **N studies** | **Coefficient** | **SE** | **95% CI** | ***p-*value** | **R^2^** |
| Year of publication | 10 | 0.0100 | 0.0504 | (-0.0887 - 0.1087) | 0.8427 | 0.00% |
| Sample size | 10 | 0.0001 | 0.0001 | (-0.0001 - 0.0002) | 0.3135 | 0.00% |
| Quality | 10 | -0.4305 | 0.5486 | (-1.5057 - 0.6447) | 0.4326 | 0.00% |
| Age | 7 | 0.0864 | 0.0540 | (-0.0195 - 0.1922) | 0.1099 | 30.47% |
| Sex Ratio (F:M) | 9 | -0.4562 | 0.6537 | (-1.7373 - 0.8249) | 0.4852 | 0.00% |


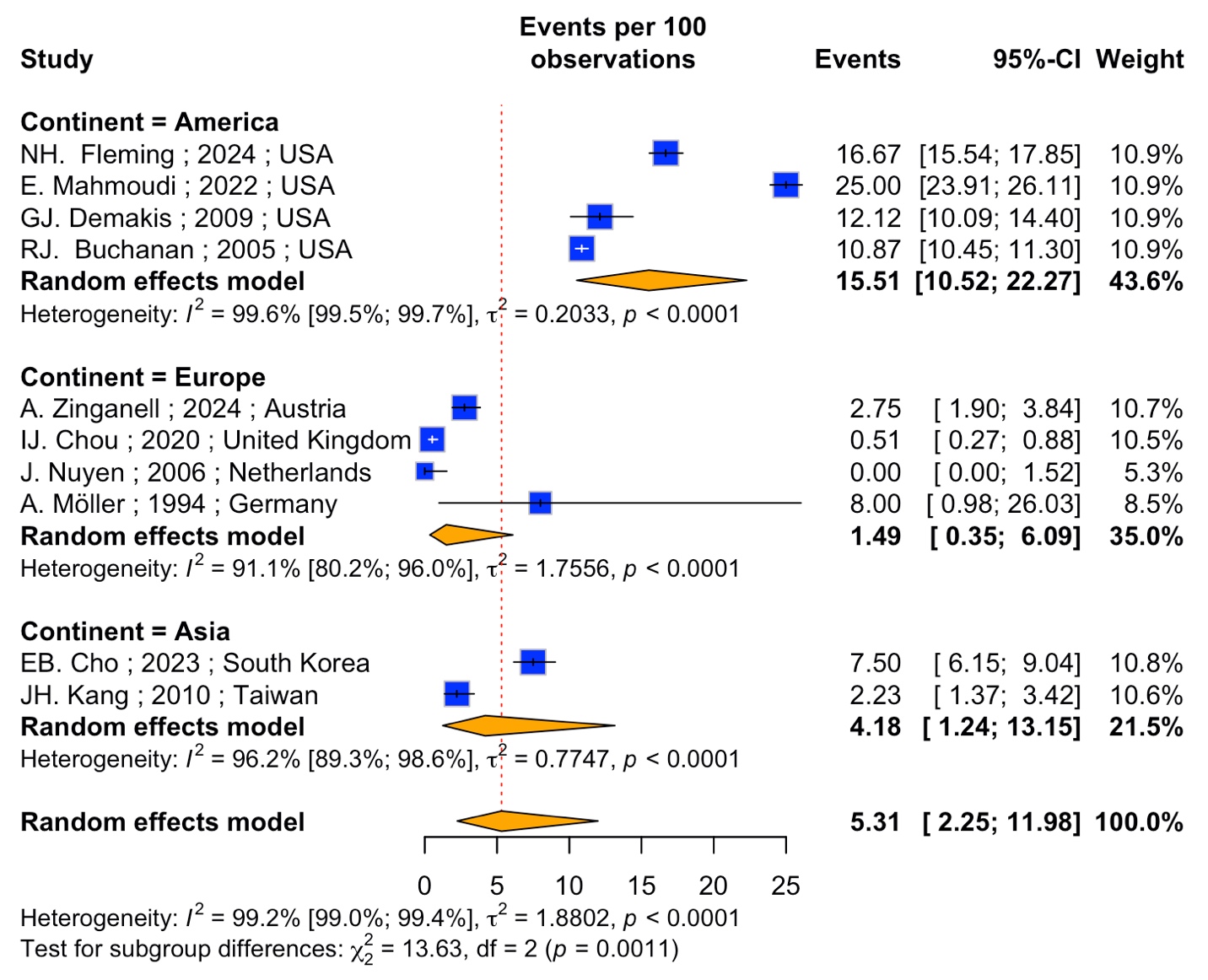


**Figure S1.** Pooled prevalence of dementia in MS patients with subgroup analysis based on the continent


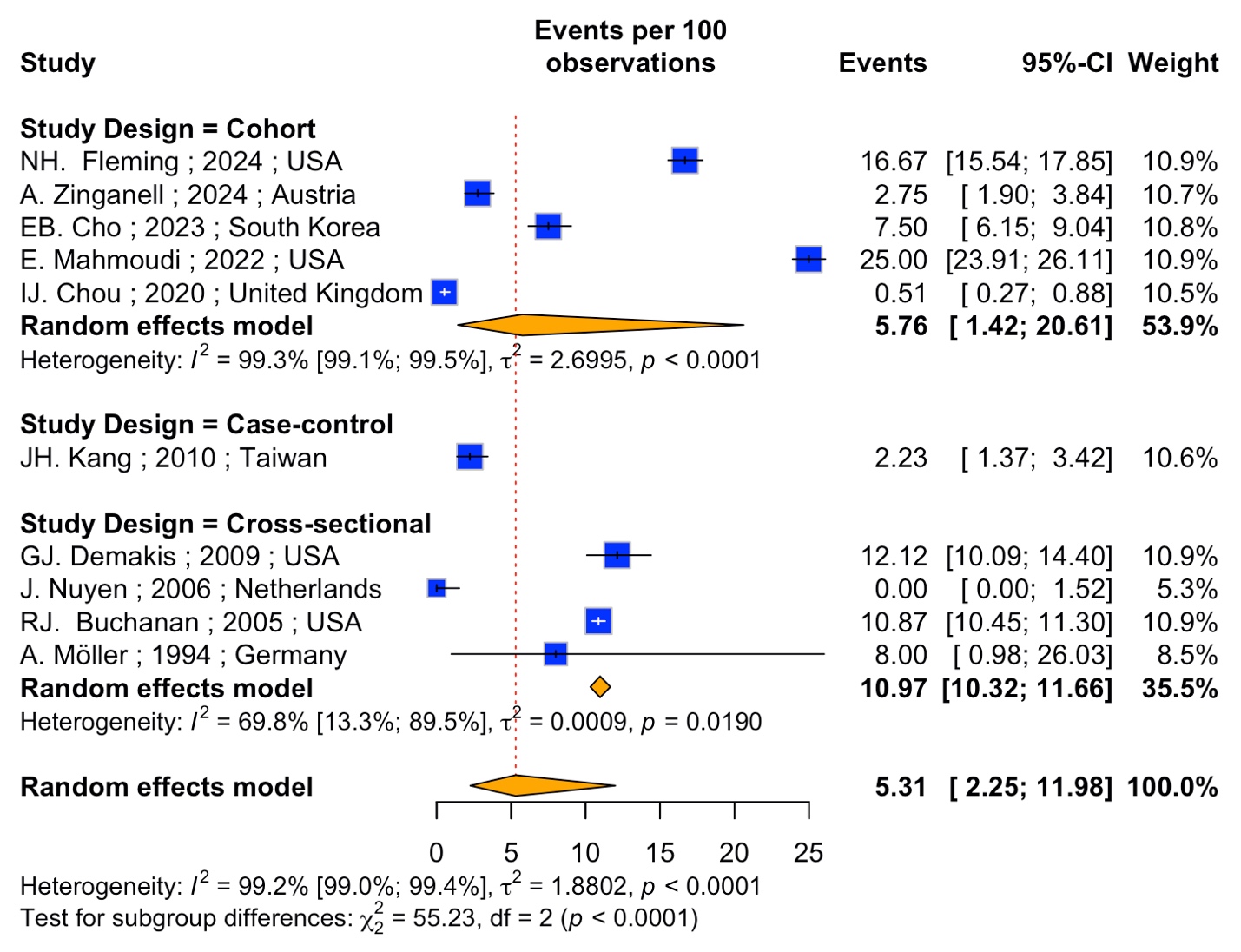


**Figure S2.** Pooled prevalence of dementia in MS patients with subgroup analysis based on the study design


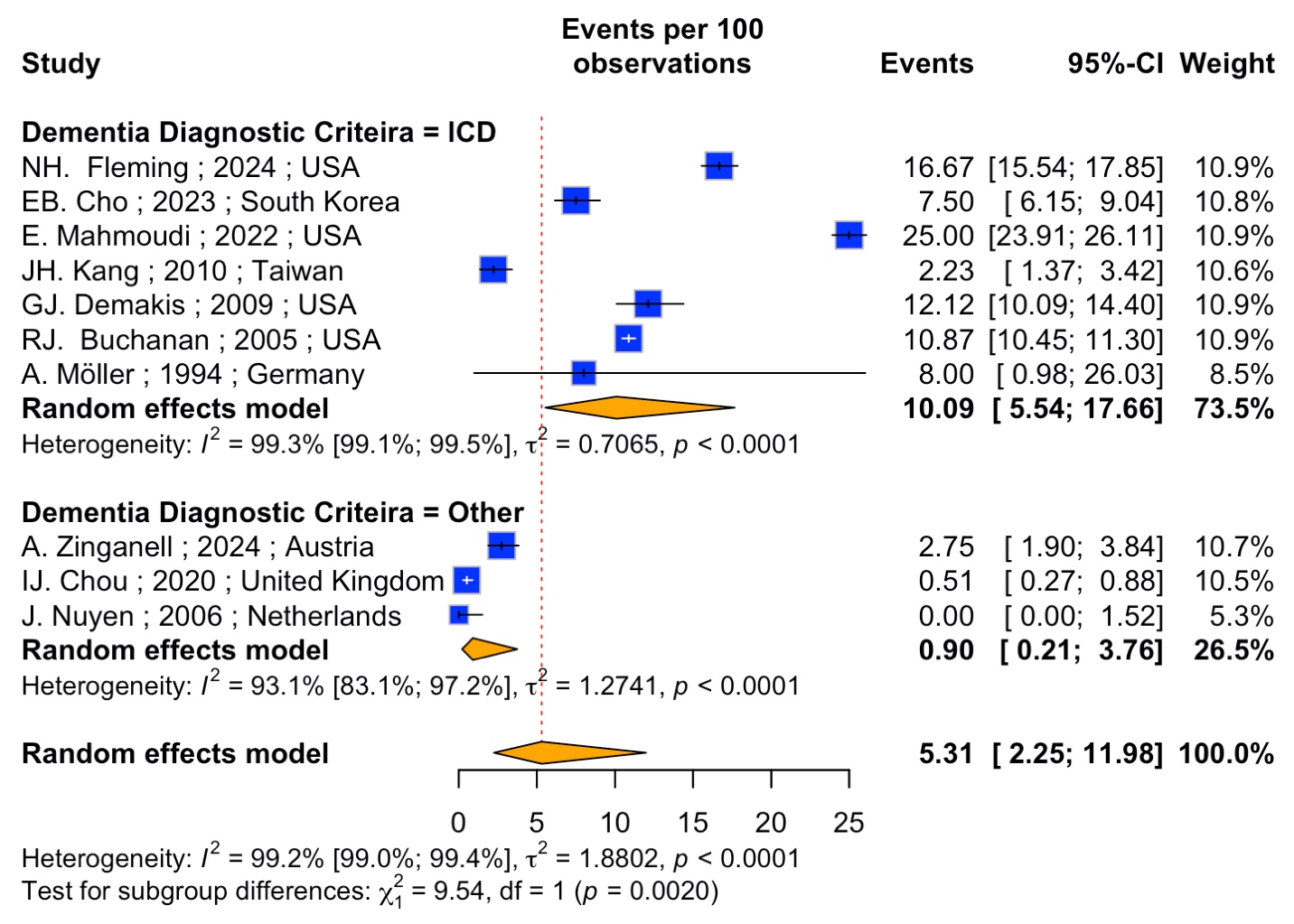


**Figure S3.** Pooled prevalence of dementia in MS patients with subgroup analysis based on the dementia diagnostic criteria


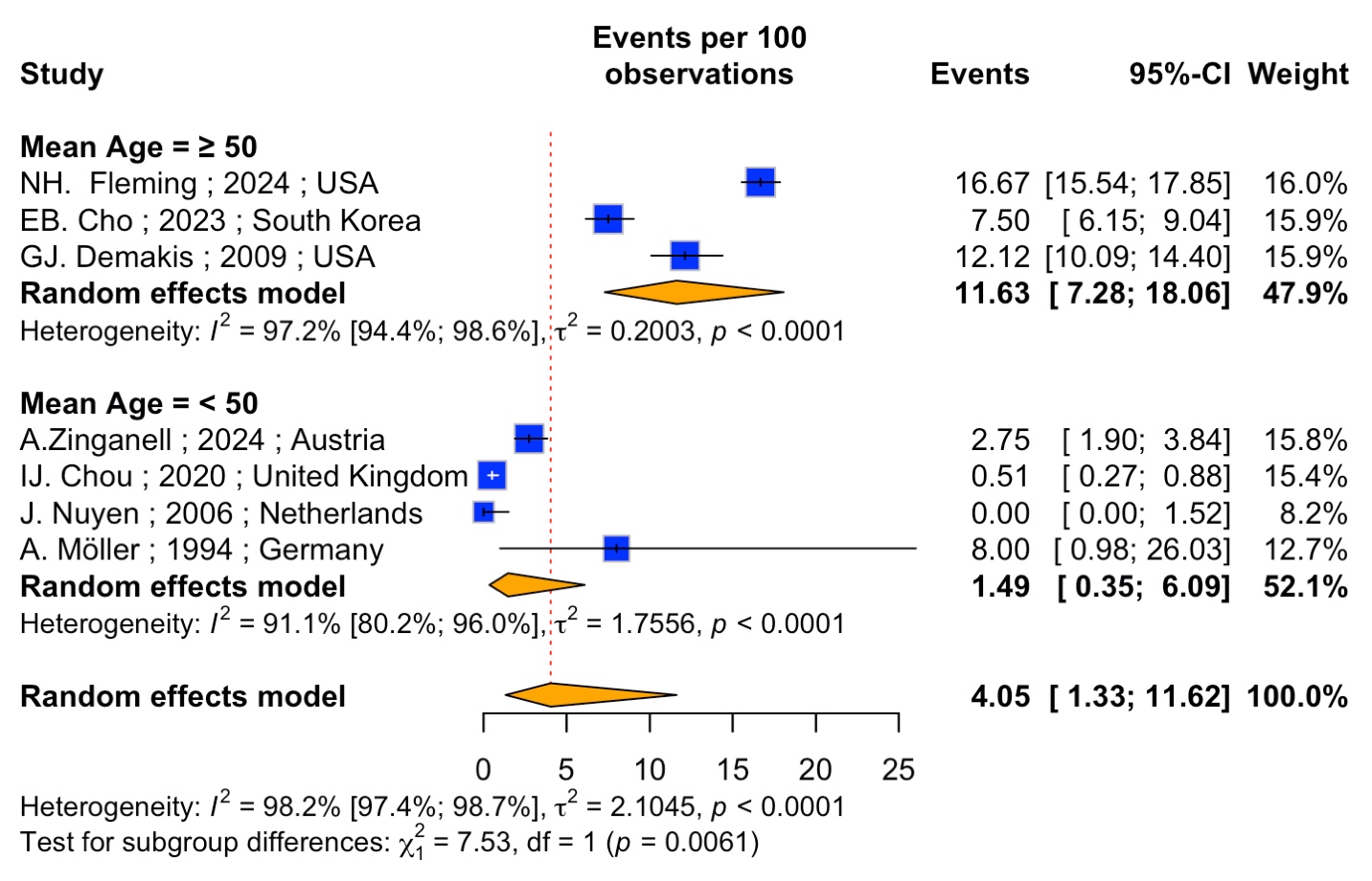


**Figure S4.** Pooled prevalence of dementia in MS patients with subgroup analysis based on the mean age of patients in included studies


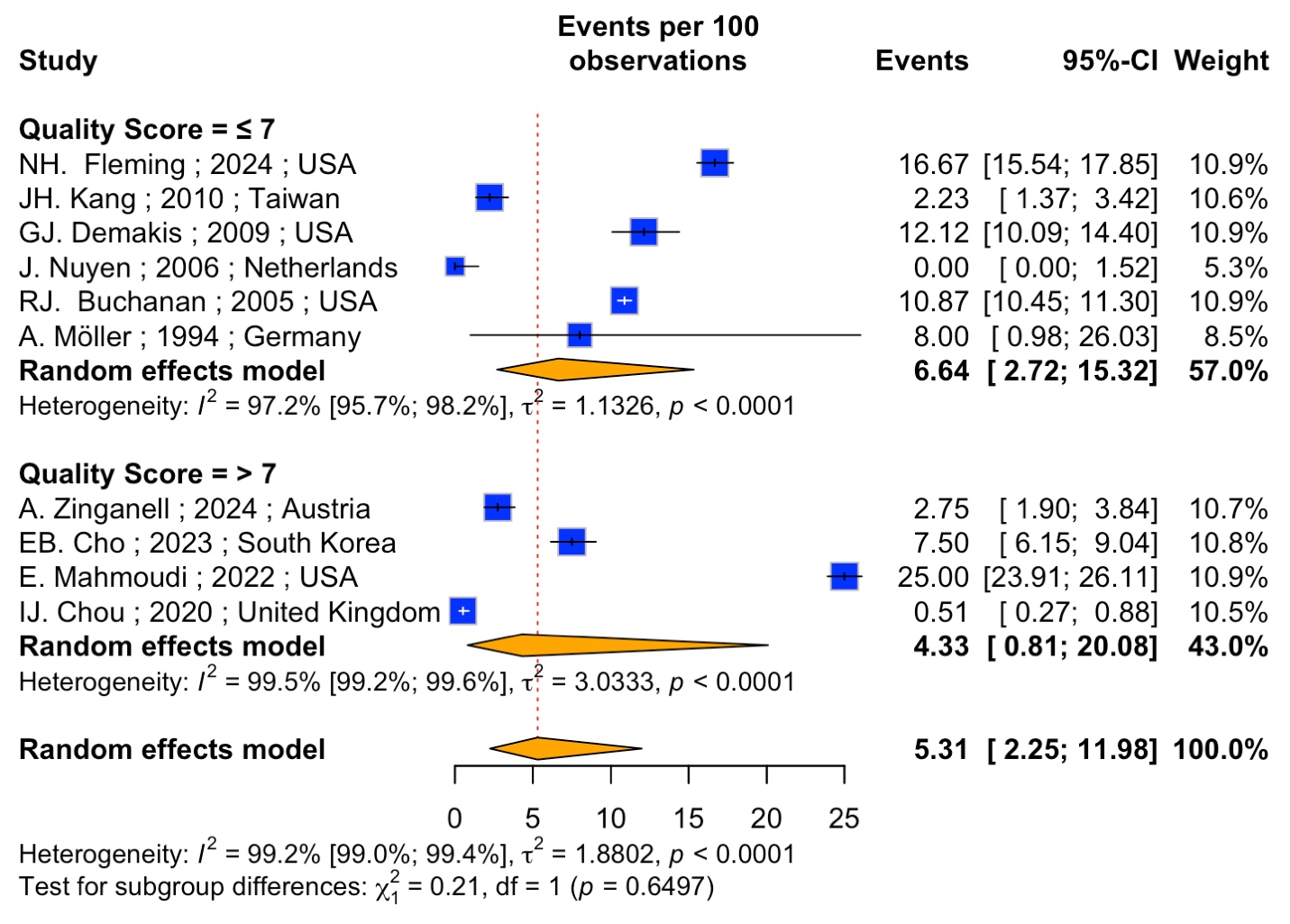


**Figure S5.** Pooled prevalence of dementia in MS patients with subgroup analysis based on the study quality


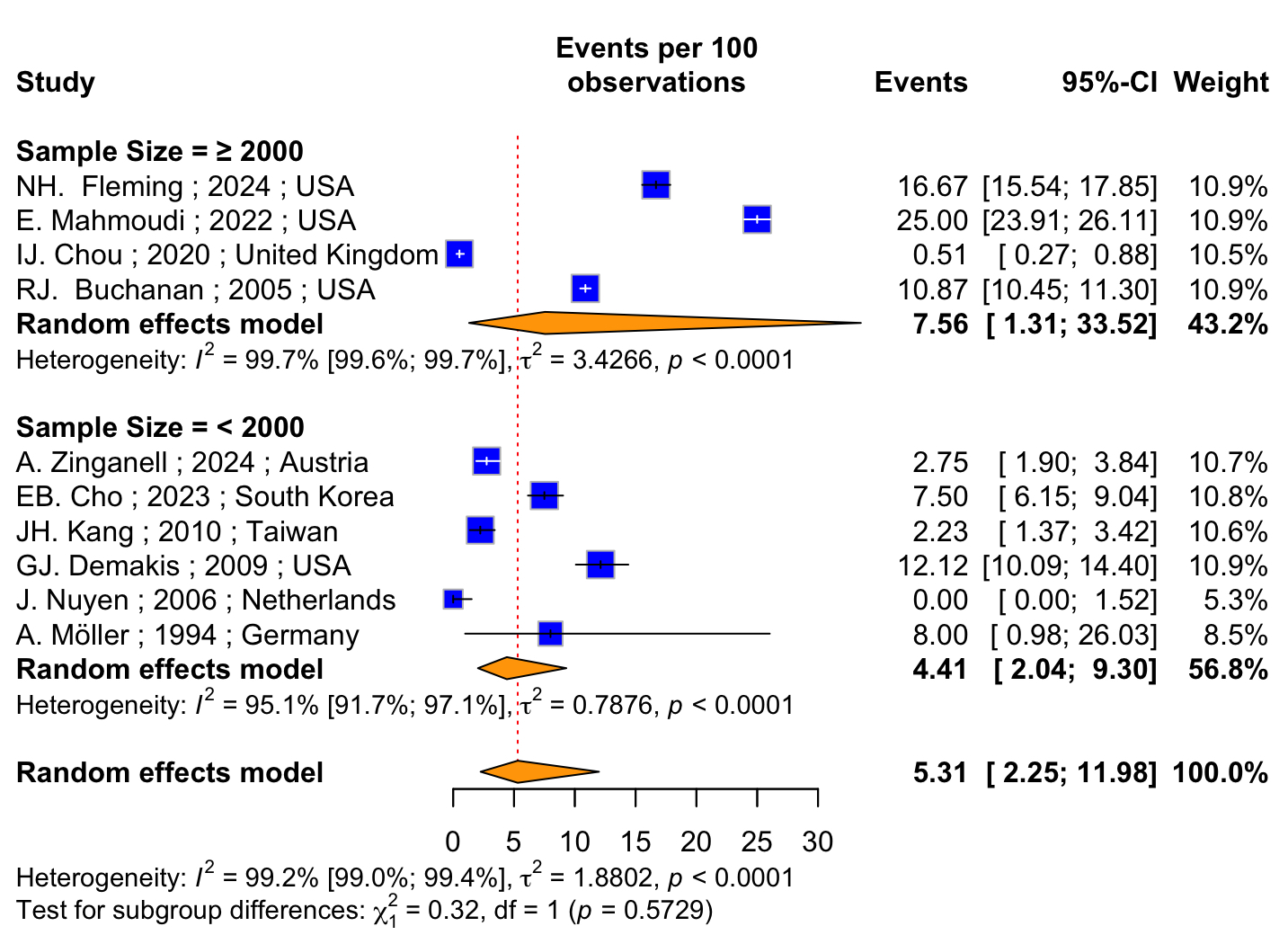


**Figure S6.** Pooled prevalence of dementia in MS patients with subgroup analysis based on the sample size


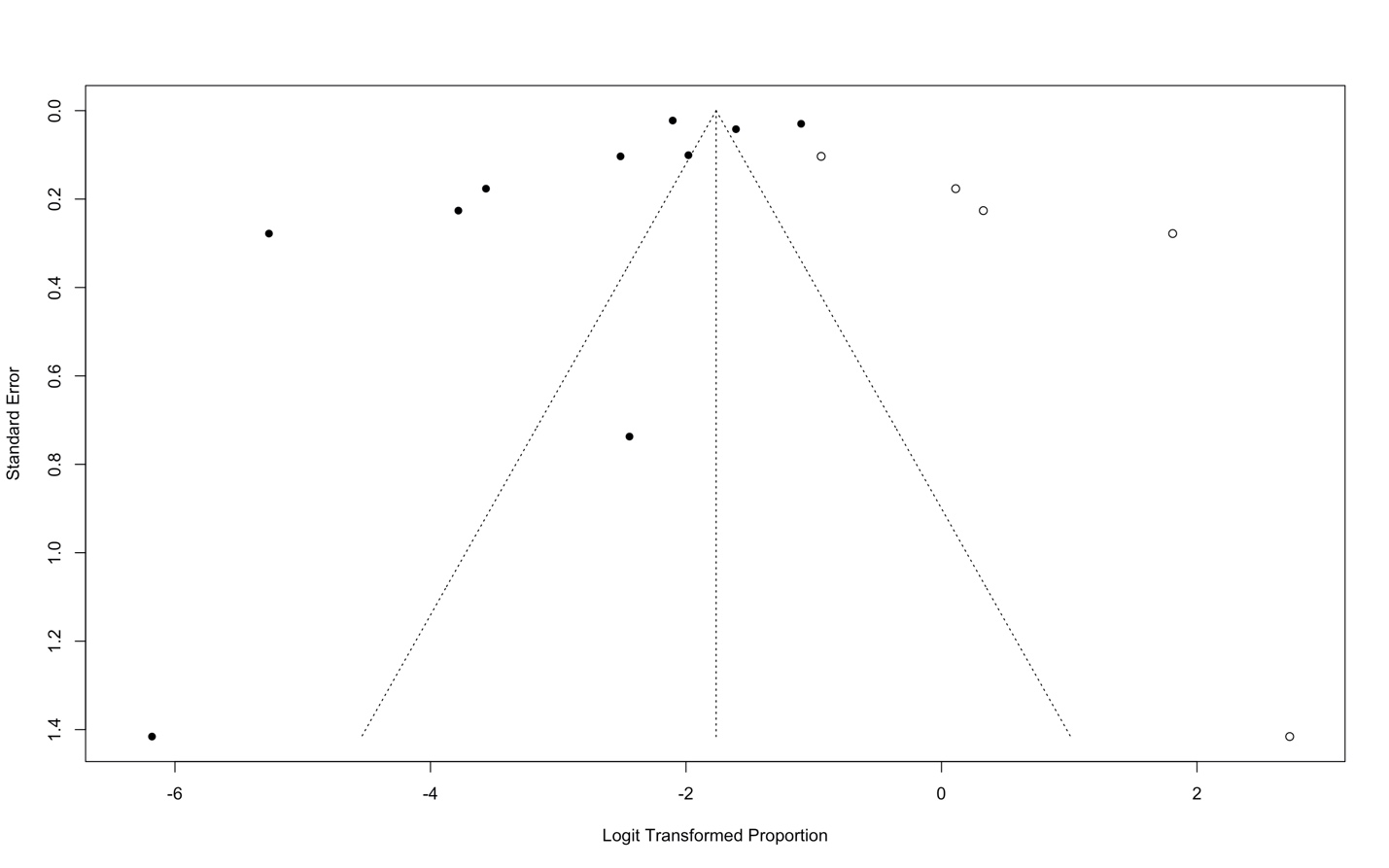


**Figure S7.** Trim-and-fill analysis adjusted for publication bias by imputing five missing studies


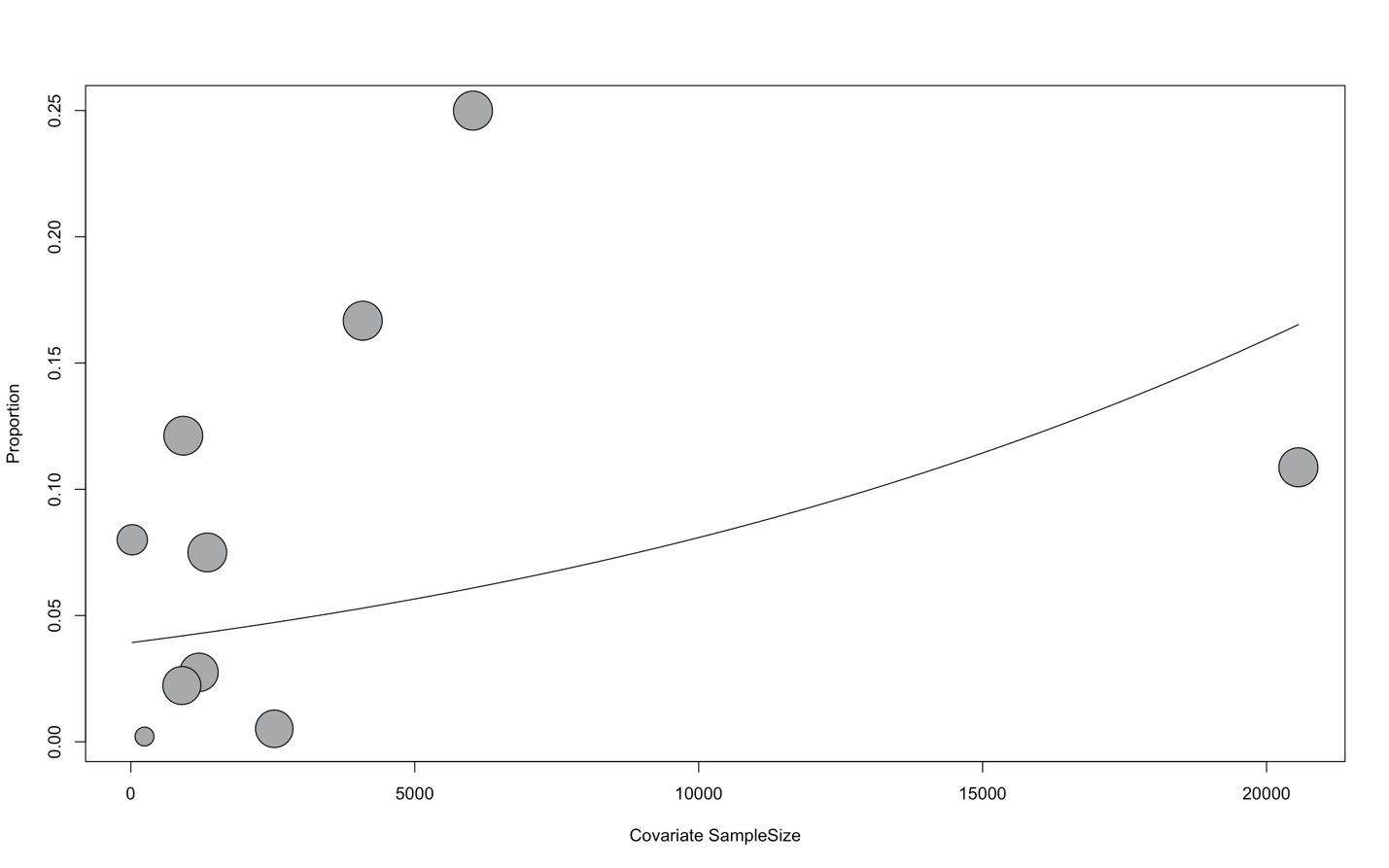


**Figure S8.** Meta-Regression analysis of the impact of sample size on the pooled prevalence of dementia in MS patients


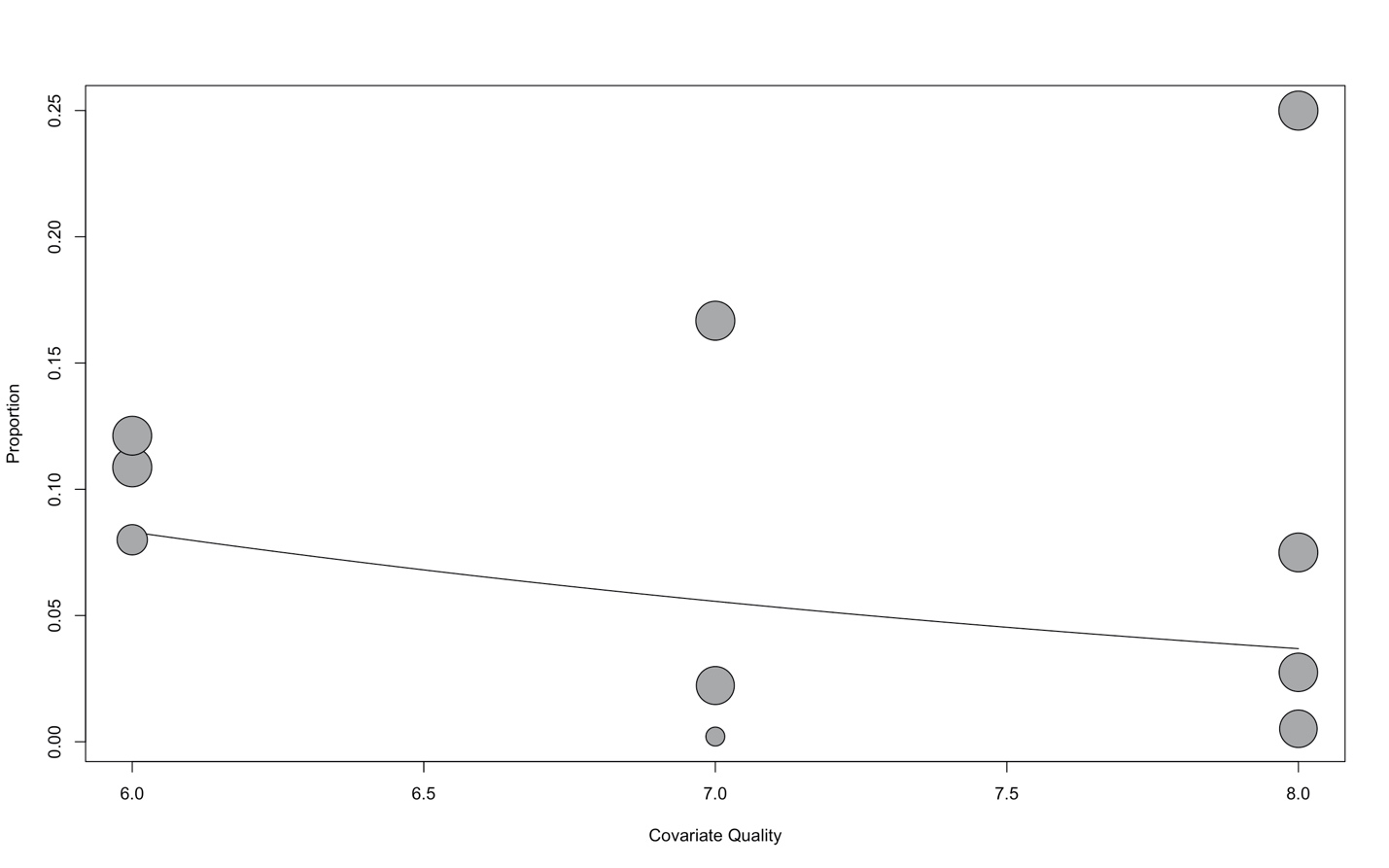


**Figure S9.** Meta-Regression analysis of the impact of quality of the included studies on the pooled prevalence of dementia in MS patients


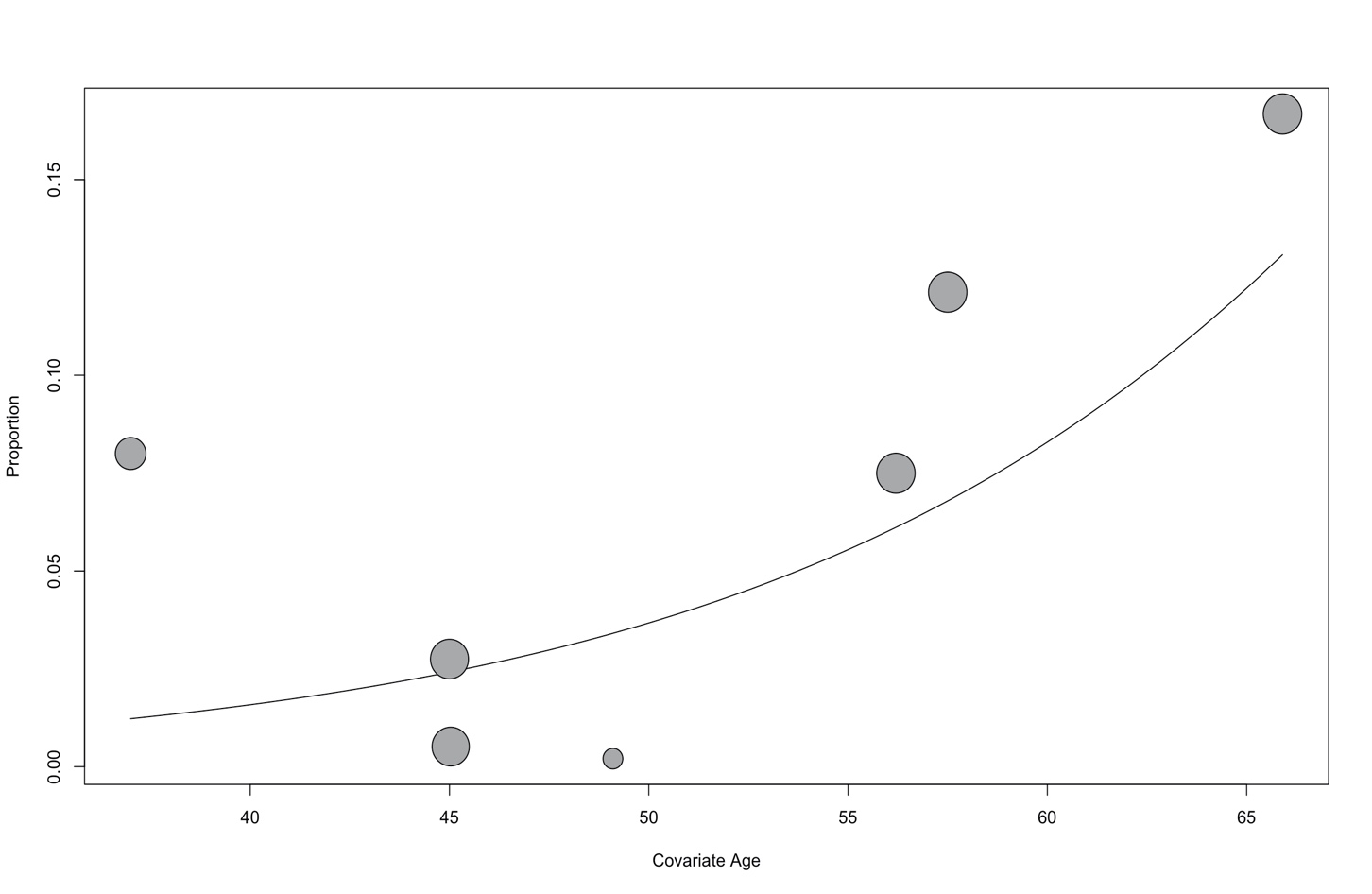


**Figure S10.** Meta-Regression analysis of the impact of age on the pooled prevalence of dementia in MS patients


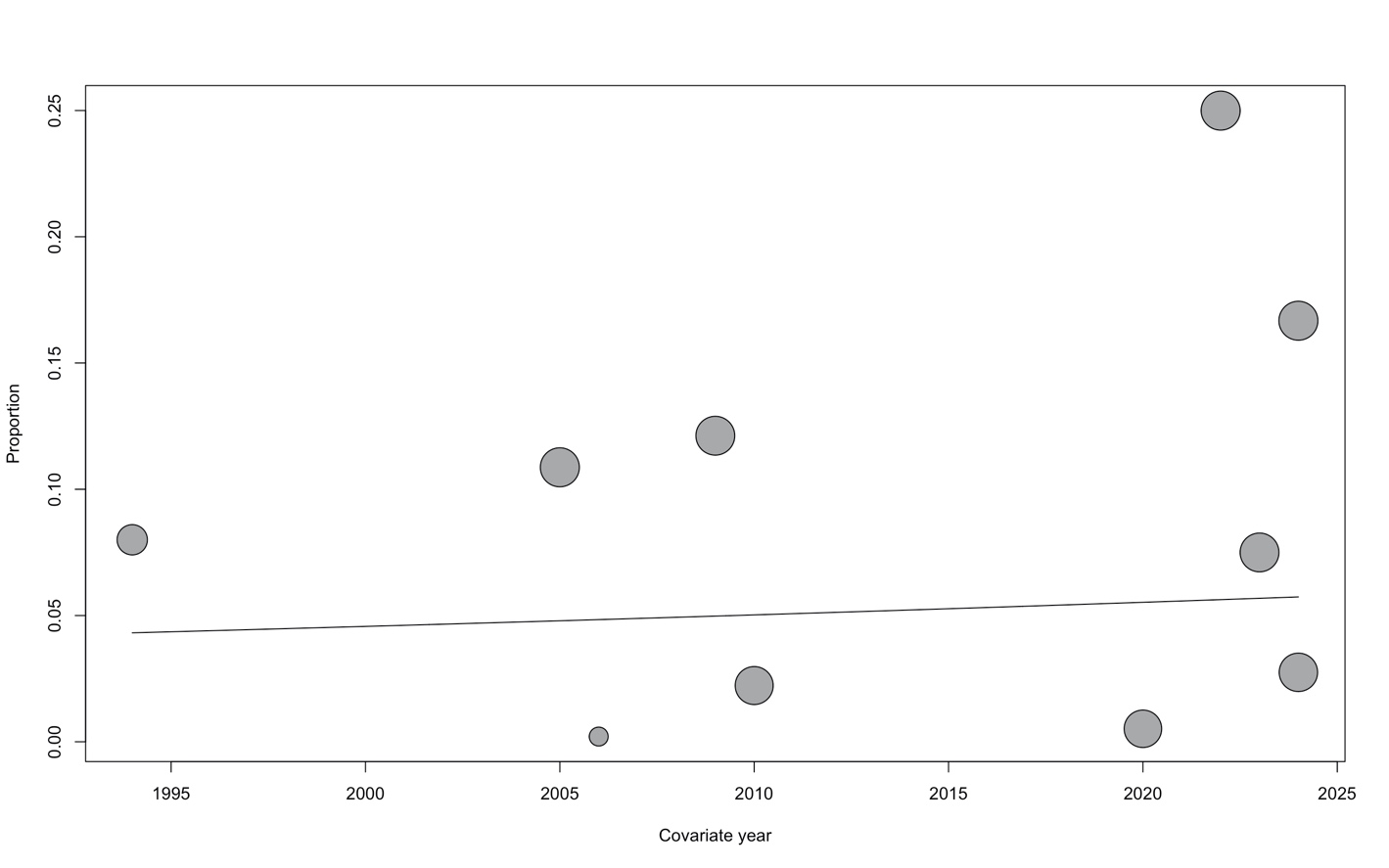


**Figure S11.** Meta-Regression analysis of the impact of publication year on the pooled prevalence of dementia in MS patients


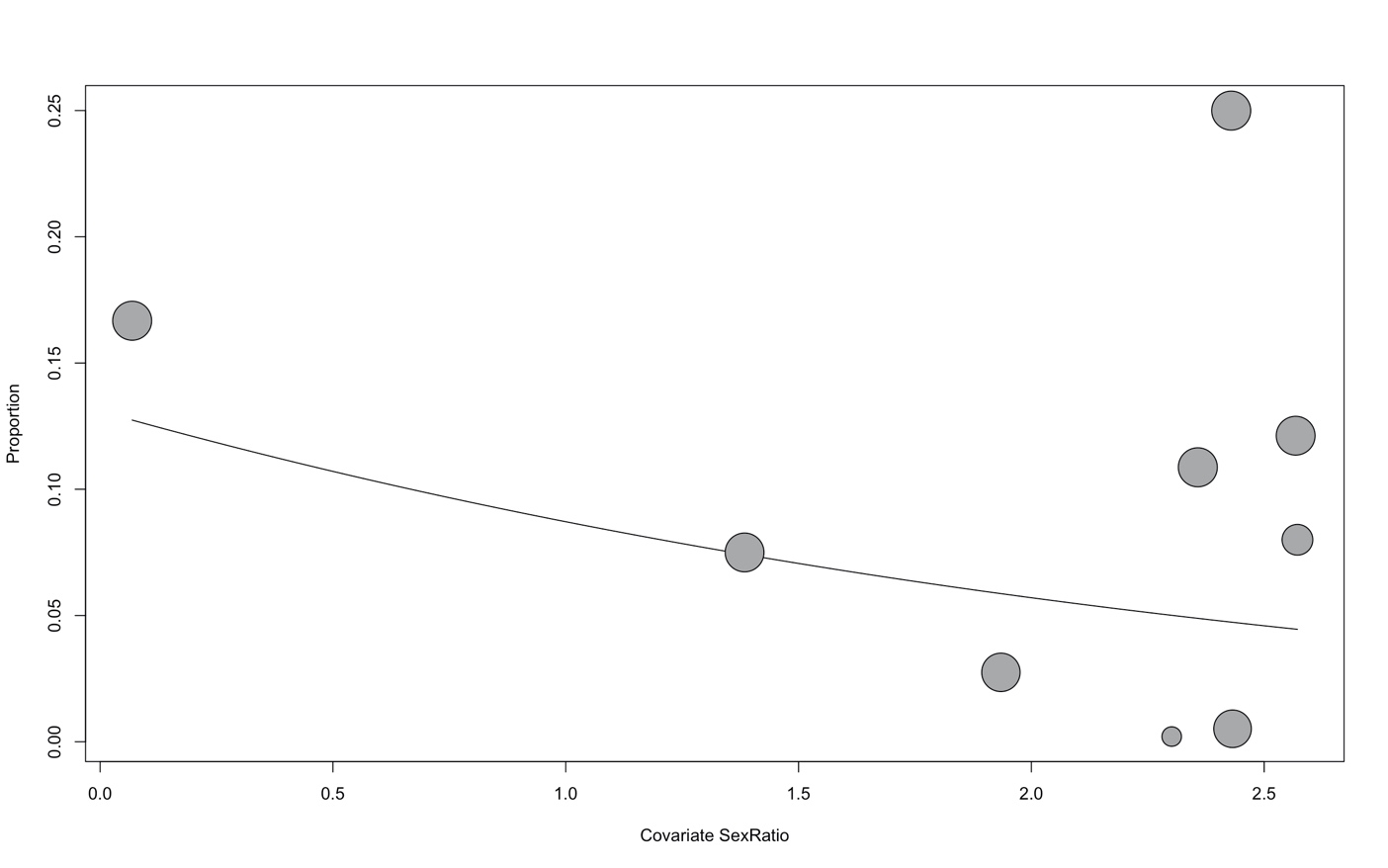


**Figure S12.** Meta-Regression analysis of the impact of sex ratio(F:M) on the pooled prevalence of dementia in MS patients
